# Systematic reviews in public health: Exploring challenges and potential solutions

**DOI:** 10.25646/6504

**Published:** 2020-06-04

**Authors:** Stefan K. Lhachimi

**Affiliations:** University of Bremen, Institute for Public Health and Nursing Research

Systematic reviews (SR) utilizing meta-analysis of randomized controlled trials (RCTs) are a foundation block of evidence-based medicine, with Cochrane reviews often being considered the gold standard. Systematic reviews of public health interventions (SR-PHI) are certainly desirable and conducted with increased frequency [1]. Nevertheless, public health interventions (PHI) differ substantially from clinical interventions but a precise demarcation remains elusive. Commonly, PHIs are some kind of public policy or behavioural intervention that aims to protect or improve health at the population level; not merely a primarily clinical intervention that has ‘public health relevance’ due to disease burden or patient numbers (e.g. vaccination). Drawing from a set of published and ongoing Cochrane reviews on fiscal policies employed as public health interventions – unconditional cash transfers (UCT) [2, 3] and taxation of high caloric foods (‘sin taxes’) [4–6] – we will present several challenges of SR-PHIs.

First, conducting SR-PHIs is time consuming: For SRs published by the Cochrane Public Health Group in the previous two years, the median duration between publishing the protocol and publishing the review was approx. 56 months (range: approx. 22–78 months). Usually, the search must be broader in terms of types of literature (in particular grey literature or policy papers) and search strategy. Recent research on abbreviated search strategies suggests, however, that the scope for reducing the search effort for SR-PHIs is currently lower than for clinical interventions [7].

Second, primary studies used for SR-PHIs are often not randomized. Even in cases using RCT designs, studies lack important characteristics that ensure a low risk of bias. For example, behavioural interventions cannot be blinded and contamination often cannot be avoided. A newly developed risk of bias tool (ROBINS-I) aims to mitigate this challenge by potentially allowing to upgrade certain non-random studies to ‘moderate quality’ [8].

Third, primary studies for PHIs are less standardized: Evidence-based medicine explicitly aims for replication of previous findings to increase robustness of the evidence base. In contrast, public health studies differ often in their precise intervention-specification, and often no agreed standards exist on primary endpoints or minimum significant differences.

Fourth, transferability of the findings of an SR-PHI to a different setting can be unclear: An understudied aspect of transferability of a given PHI is the political feasibility in a different polity (e.g. are unconditional cash transfers truly acceptable for the general population). Another challenge is that jurisdictions/population differs substantially in their health and behavioural profile. Hence, applying particular interventions can yield very different outcomes in terms of magnitude [9]. Tools originating from quantitative health impact assessment are an option to quantify prospectively the consequences of a public health population for a particular intervention [10].

In conclusion, the relative complexity and duration of SR-PHIs are in stark contrast to the need of giving well-timed policy advice. For some of these outlined challenges methodological work is already ongoing. Nevertheless, designer of primary studies for PHIs must pay more attention to potential inclusion in a future SR by standardising interventions and endpoints.
